# Case Report: Robotic pylorus-preserving pancreatoduodenectomy for periampullary rhabdomyosarcoma in a 3-year-old patient

**DOI:** 10.3389/fsurg.2024.1284257

**Published:** 2024-02-19

**Authors:** Zijian Liang, Menglong Lan, Xiaogang Xu, Fei Liu, Boyuan Tao, Xinxing Wang, Jixiao Zeng

**Affiliations:** Department of Pediatric Surgery, Guangzhou Women and Children's Medical Center, Guangzhou Medical University, Guangzhou, Guangdong, China

**Keywords:** robotic pancreatoduodenectomy, periampullary neoplasm, rhabdomyosarcoma, child, case report

## Abstract

Periampullary neoplasm is rare in pediatric patients and has constituted a strict indication for pancreatoduodenectomy (PD), which is a procedure sporadically reported in the literature among children. Robotic PD has been routinely performed for periampullary neoplasm in periampullary neoplasm, but only a few cases in pediatric patients have been reported. Here, we report the case of a 3-year-old patient with periampullary rhabdomyosarcoma treated with robotic pylorus-preserving PD and share our experience with this procedure in pediatric patients. A 3-year-old patient presented with obstructive jaundice and a mass in the pancreatic head revealed by imaging. A laparoscopic biopsy was performed. Jaundice progressed with abdominal pain and elevated alpha-amylase leading to urgent robotic exploration in which a periampullary neoplasm was revealed and pathologically diagnosed as rhabdomyosarcoma by frozen section examination. After pylorus-preserving PD, we performed a conventional jejunal loop following a child reconstruction, including an end-to-end pancreaticojejunostomy, followed by end-to-side hepaticojejunostomy and duodenojejunostomy. Delayed gastric emptying (DGE) presented with increasing drain from the nasogastric tube (NGT) a week after the surgery and improved spontaneously within 10 days. In a 13-month follow-up until the present, our case patient recovered well without potentially fatal complications, such as pancreatic fistula. Robotic PD in pediatric patients was safe and effective without intra- or postoperative complications.

## Introduction

Pancreatoduodenectomy (PD) was first introduced in a case report by Whipple et al. in 1935 (1). The pylorus-preserving modification was described by Traverso and Longmire in 1978 (2). Currently, PD is routinely performed for pancreatic head neoplasms. Parallel to the development of surgical instruments and the introduction of minimally invasive surgery, laparoscopic and robotic PD has become a common procedure and has been reported to have better clinical outcomes compared with open surgery (3–6). However, laparoscopic and robotic PD for pediatric patients has only been sporadically reported in the literature (7–9). Here, we report the case of a 3-year-old patient with periampullary rhabdomyosarcoma treated with robotic pylorus-preserving pancreatoduodenectomy (PPPD) and share our experience with this procedure in pediatric patients. We present the following case in accordance with the SCARE criteria (10).

## Case description

A 3-year-old Chinese patient was referred to our hospital because of white, clay-like stools and a 34 mm  × 29 mm  × 34 mm mass in the lower part of the biliary tract and pancreatic uncinate process revealed by ultrasonic examination. The patient had intermittent abdominal pain and did not have any signs of fever, abdominal distention, emesis, or skin itching in the previous 20 days. No eventful history was found after a detailed consultation. Physical examination revealed slight conjunctival jaundice without any abdominal abnormal signs. Laboratory tests revealed elevated liver enzymes [alanine aminotransferase (ALT) 455 U/L, aspartate aminotransferase (AST) 366 U/L, gamma-glutamyl transferase (GGT) 1,637 U/L] and obstructive jaundice with total bilirubin of 74.2 µmol/L and direct bilirubin of 52.6 µmol/L. Carbohydrate antigen-199 (Ca-199) was 3,752.91 IU/ml and neuron-specific enolase (NSE) was 56.21 ng/ml.

**Figure 1:**
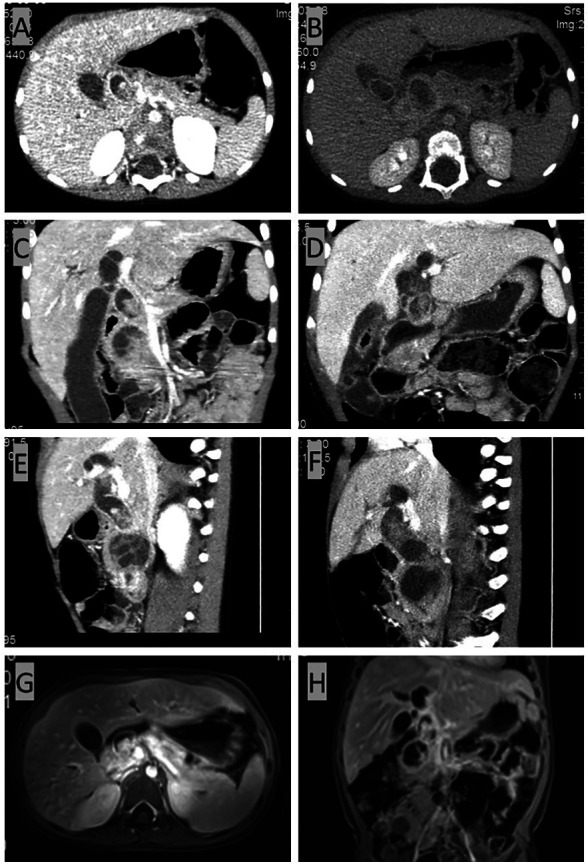
CT and MR results before radical surgery. (**A,C,E**) CT scan of the abdomen and pelvis with intravenous contrast agent showing an ill-defined 2.9 cm  × 2.8 cm mass with delayed enhancement of the uncinate process with proximal dilatation. (**G**,**H**) MR imaging showing a 2.6 cm × 3.6 cm × 2.1 cm mass originating from the common bile duct (CBD), presenting an equal signal on T1WI and an equal-high signal on T2WI. The common bile duct (CBD) was plugged up completely by the mass, resulting in the dilatation of the proximal biliary system. The dilatation of the common bile duct (CBD) reached 2.2 cm in diameter, and the pancreatic duct also presented a slight dilation of 0.1 cm. (**B**,**D**,**F**) CT scan for reevaluation showing the enlarged mass.

Computed tomography (CT) scan of the abdomen and pelvis with an intravenous contrast agent revealed an ill-defined 2.9 cm × 2.8 cm mass with delay enhancement in the uncinate process and common bile duct (CBD) and dilation in the biliary system (Figures 1A,C,E). Magnetic resonance (MR) imaging showed a 2.6 cm × 3.6 cm × 2.1 cm mass assumed to originate in the CBD, presenting an equal signal on T1WI and an equal-high signal on T2WI, which caused a thorough cutoff of the CBD (Figures 1G,H). Proximal biliary tract diameter was 2.2 cm while the MPD measured 0.1 cm. Positron emission tomography (PET) did not show any extrapancreatic disease.

**Figure 2:**
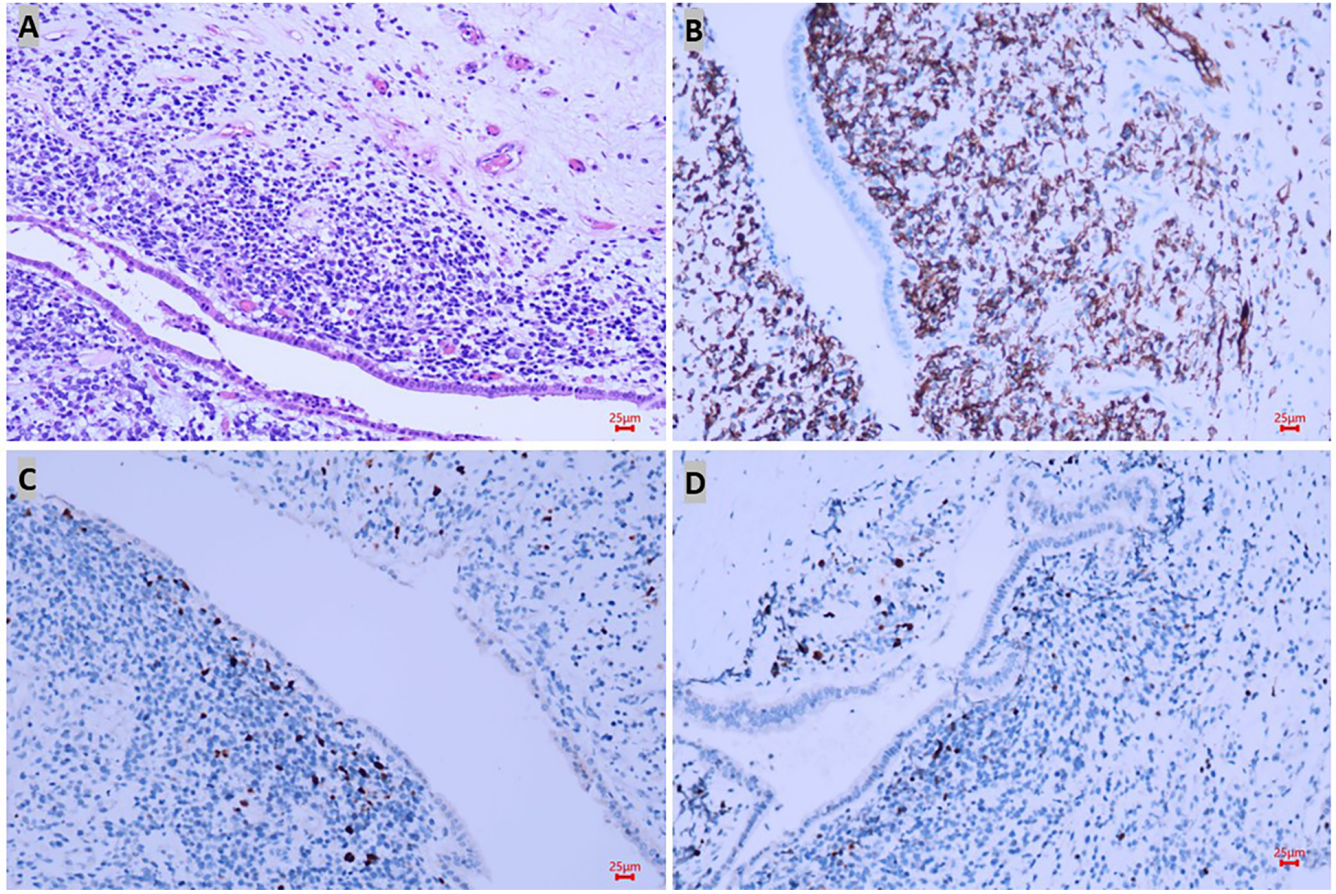
Pathological results confirmed the diagnosis of rhabdomyosarcoma. In hematoxylin–eosin staining, the lesion was located under the epithelium and formed a neoplastic layer parallel to the epithelium with small round atypic cells with obvious heteromorphism (**A**), and positive markers such as desmin (**B**), myoD1 (**C**), and myogenin (**D**) staining confirmed the diagnosis of embryonal RMS originated in the ampulla.

As malignant pancreatic tumors are rare in children, the diagnosis with the pathological result would be the key for further management. A laparoscopic biopsy with cholecystostomy drainage was performed. The cholecystostomy for biliary drainage was successful as the level of bilirubin decreased; however, the biopsy was negative. While waiting 1 week for the pathological result, we performed another CT scan, which showed that the mass had enlarged to 4.4 cm × 4.3 cm (Figures 1B,D,F) while alpha-amylase had risen to 549 U/L. Surgery was decided upon due to the rapid evolution of tumor size and increased signs of abdominal pain. After malignancy was confirmed by frozen section examination, a PPPD with an end-to-end pancreatojejunostomy followed by end-to-side hepaticojejunostomy and duodenojejunostomy was performed. Liver enzymes, bilirubin, alpha-amylase, and Ca-199 gradually decreased to normal levels within one week after the surgery. Amylase levels in the drainage fluid, tested every 3 days, were negative. The drains were removed when the daily drains were <20 ml and lasted for more than 3 days. The nasogastric tube (NGT) was removed 17 days postoperatively as the drain had dropped to under 20 ml in the last 3 days, and the patient was able to maintain unlimited oral intake in 21 days postoperatively. Delayed gastric emptying (DGE) was defined as Grade B according to the International Study Group of Pancreatic Surgery (11). Pathological examination with desmin, myogenin, and myoD1 staining confirmed the diagnosis of embryonal RMS originating from the ampulla (Figure 2). Because of negative surgical margins and the absence of lymph node involvement, the patient was classed as low risk and was recommended to undergo chemotherapy after fully recovering from surgery. At 13 months of follow-up, the patient has tolerated chemotherapy well and has shown no signs of recurrence.

### Surgical procedure

**Figure 3:**
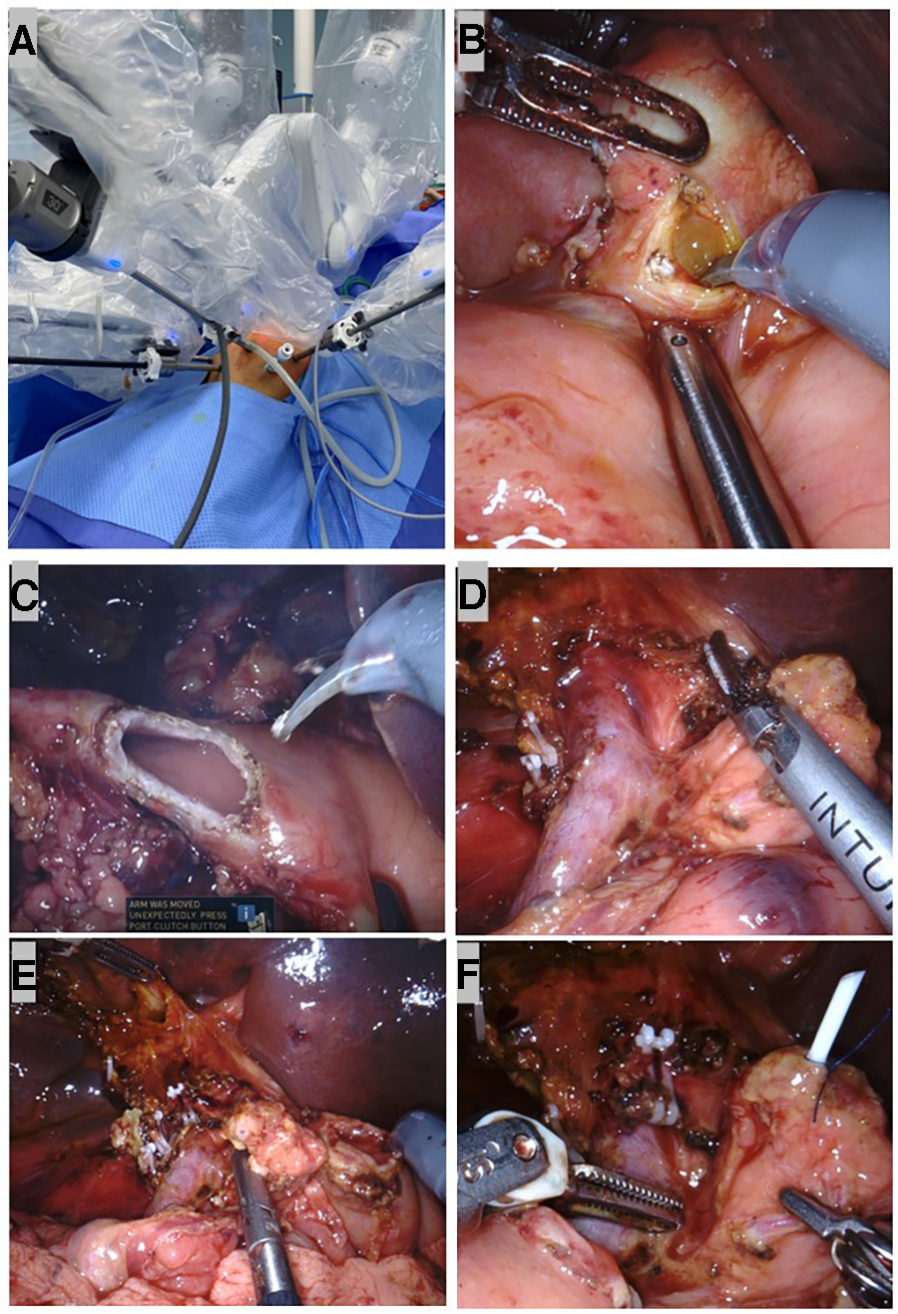
Robotic dissection in pylorus-preserving pancreatoduodenectomy (PPPD). (**A**) Port set up during surgery. (**B**) A neoplasm resembled a cluster of grapes inside the common bile duct (CBD). (**C**) The duodenum was transected at 2 cm distal to the pylorus. (**D**) The skeletonized SMV/SMA and dissected retroperitoneum with peripancreatic soft tissue and the nerve plexuses removed. (**E**) After the specimen was taken out, a stump of the common hepatic duct, pancreas, and duodenum. (**F**) A 3F ureteral stent tube was inserted into the MPD as an internal stent and fixed to the MPD with 4-0 Prolene.

The patient was placed in the supine position. A five-port approach is shown in Figure 3A with the da Vinci Xi Surgical System. After creating a pneumoperitoneum (8 mmHg), we placed a 8 mm trocar at the umbilicus. Next, the remaining 8 mm robotic arm ports were inserted under safe vision. The first robotic arm (R1) was placed along the left midclavicular line crossing with the transverse umbilical line. The second robotic arm (R2) was placed along the right midclavicular line crossing 2 cm below the transverse umbilical line. The third robotic arm (R3) was placed under the costal margin crossing with the right anterior axillary line. Another 5 mm trocar was placed for the assistant surgeon to control suction and pass sutures.

A brief view of the abdominal cavity showed no metastasis lesions, and the CBD was dilated to a maximum diameter of 2 cm. The gastrocolic ligament was divided using the harmonic scalpel, and the stomach was lifted upward with two transabdominal stay sutures so that the enlarged pancreatic head was exposed. Next, we performed a choledochotomy where a neoplasm that resembled a cluster of grapes was revealed (Figure 3B). Biopsy was taken from both the neoplasm in the CBD and pancreatic head for fast-frozen section examination. The neoplasm showed small round atypic cells with obvious heteromorphism within the lesion; therefore, a malignant pancreatic head tumor was suspected, and the PD procedure was indicated.

After performing a 5-0 polydioxanone suture (PDS) in the biopsy site for hemostasis and closure of the tumor, we identified the anatomy in the hepatic hilum. Next, we identified the common hepatic artery (CHA), gastroduodenal artery (GDA), portal vein (PV), and biliary system, ligated the GDA at its origin, and identified and dissected the superior margin of the pancreas. During the dissection, the superior mesenteric vein (SMV) and the branches, including the superior right colic vein, the right gastroepiploic vein, and the gastrocolic trunk, were carefully divided. Then, we moved the horizontal portion of the duodenum and the dorsal portion of the pancreatic head using the Kocher maneuver and partially divided the Treitz ligament and duodenojejunal flexure for better transection of the first jejunal loop. Following the identification and ligation of the cystic artery, the gallbladder and the CBD were dissected and removed. The duodenum was then transected distal to the pylorus (Figure 3C). Simultaneously, we performed lymph node dissection of the coeliac trunk, hepatoduodenal ligament, SMV, the right side of the superior mesenteric artery, and the lymphatic tissue behind the pancreatic head; the specimens were sent for pathological examination (Figure 3D).

Next, we began to divide the pancreas and SMV from the pancreatic head to create the pancreatic tunnel using a harmonic scalpel. Through the tunnel, the transection of the pancreatic parenchyma was made until the main pancreatic duct (MPD) was identified and cut by scissors. We found a 1.5 mm MPD in the posteriolateral portion of the pancreas. An end-to-end pancreatojejunostomy was preferred to avoid anastomotic stenosis. We dissociated the stump from the tissues around the pancreas for 2 cm, which were used later for anastomosis.

After the dissection was completed, the specimens were put into an Endo Bag to avoid tumor dissemination. The margin of the duodenum, pancreas, and dissected peripyloric lymph nodes were sent for fast-frozen section examination; a negative result supported our choice for a PPPD.

**Figure 4:**
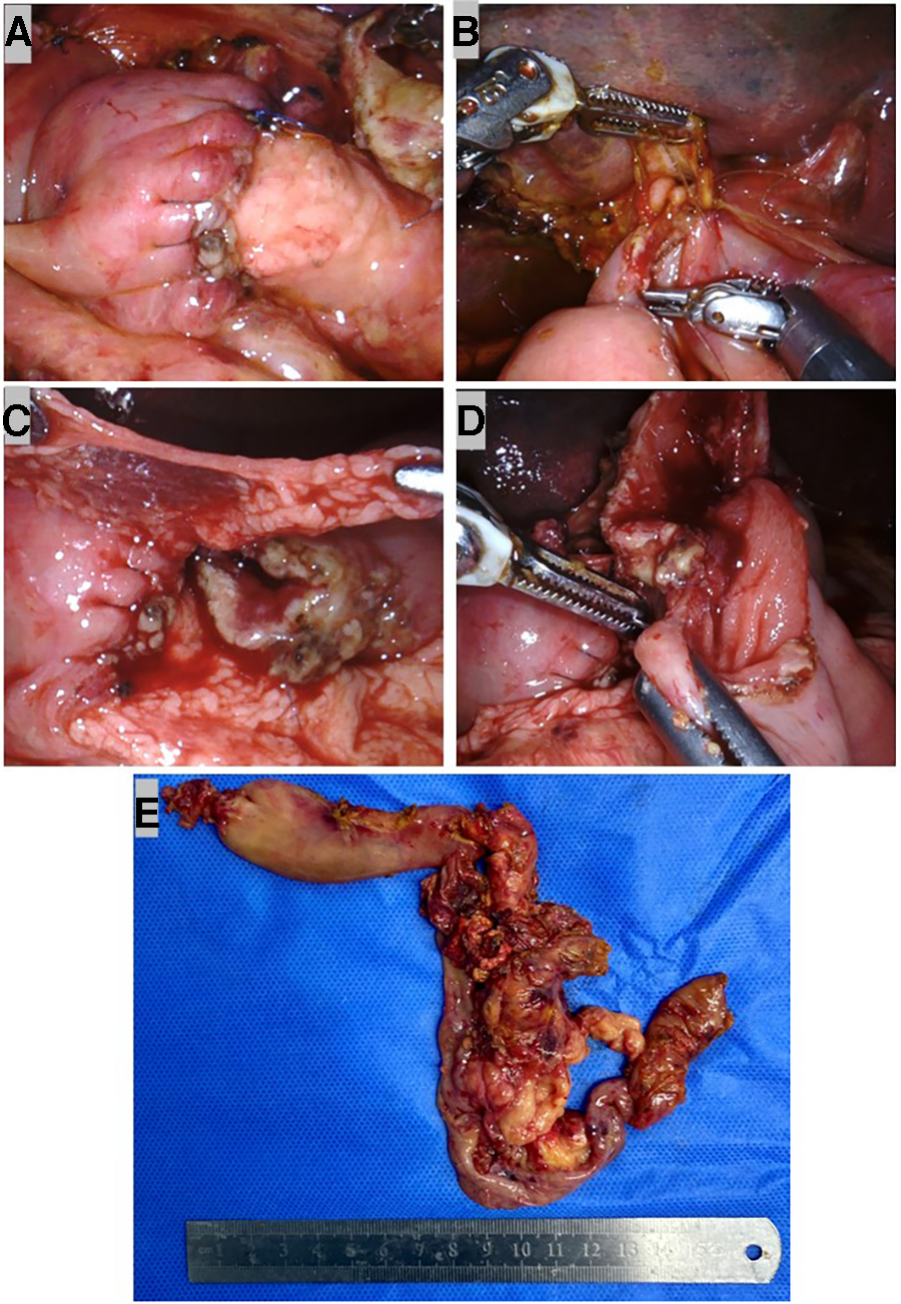
Robotic child reconstruction and specimens. (**A**) End-to-end pancreatojejunostomy. (**B**) End-to-side single layer barbed suture hepaticojejunostomy. (**C**) The distal and free portion of omentum was fixed with 5–0 Prolene suture around the pancreatojejunostomy anastomosis as a mattress. (**D**) End-to-side single-layer barbed suture duodenojejunostomy. (**E**) Specimens removed with the Endo Bag.

A child reconstruction was performed sequentially: an end-to-end pancreaticojejunostomy, followed by an end-to-side hepaticojejunostomy and duodenojejunostomy (Figure 3E). A 3F ureteral stent tube was inserted into the MPD as an internal stent and fixed with 4-0 Prolene (Figure 3F). Then a single layer of continuous suturing was performed in anterior and posterior anastomosis between the pancreatic stump and dissected jejunum, respectively. To avoid pancreatic leaks, we passed every suture through the entire layer of the jejunum and vertically into the pancreas. Finally, both ends of the sutures in the anterior and posterior walls were tightened and tied for complete coverage by the jejunal end (Figure 4A). Before the hepaticojejunostomy and duodenojejunostomy anastomosis was performed, the jejunal loop was rotated behind the mesenteric root, and hepaticojejunostomy was created where the loop was placed at the hepatic hilum without any tension. The hepaticojejunostomy anastomosis was performed in a single layer with a barbed suture, posterior and anterior, respectively (Figure 4B). The distal and free portion of the omentum was fixed with a 5-0 Prolene suture around the pancreatojejunostomy anastomosis as a mattress (Figure 4C). Then the jejunal loop was placed in an antecolic position, and end-to-side duodenojejunostomy was created. The duodenojejunostomy was performed in a single layer with a barbed suture, posterior and anterior, separately (Figure 4D). Three drains were placed close to the anastomoses. After being taken out with the Endo Bag, the specimens were dissected; these revealed a neoplasm that originated in the ampulla (Figure 4E).

## Discussion

RMS is a rare malignant tumor morphologically akin to the skeletal muscle (12). Its incidence rate is 4.5 cases per million people per year, occurring more often in children than in adults (13). In children, the most common sites include the head and neck, genitourinary tract, and extremities; the retroperitoneum or biliary tract has only been sporadically reported in the literature (14, 15). The disease presents differently according to the involved site. Diagnosis is usually made by direct biopsy (12, 14–16). Multidisciplinary treatments such as chemotherapy, radiation, and surgery have helped improve the survival rate to 70% over the past 30 years (17).

Our intraoperative decision to perform PPPD was fully justified as our patient had no direct invasion into the surrounding organs or any peripyloric lymph node metastases, as confirmed by a frozen section examination (4, 18). The rationale behind PPPD was to reduce complications following gastric resection, such as diarrhea, dumping, ulceration, and bile reflux gastritis (18, 19). Other reported advantages are shorter surgical time (4, 19), less intraoperative blood loss (4, 19) with similar complications (4, 19), reoperation (4), mortality (4), and cumulative survival rates (4) compared with standard PD. However, the effectiveness of PPPD has been doubted since it was practiced clinically for the common postoperative complication DGE and a compromised resection that may fail to reach R0 resection. In our case, we found DGE as Grade B and performed R0 resection successfully, and lymph nodes were confirmed by pathological results (11). DGE is one of the most common postoperative complications in PPPD and is thought to be caused by damage to the gastroenteric nervous system during surgery (18). DGE is reported to be transient and will be recovered with gastric suction for more than 10 days (18). In our case, the drain from the NGT gradually decreased, and meals became tolerated 17 days after the surgery, which was consistent with the literature. An early clinical report by Roder et al. (5) argued that an incomprehensive resection in PPPD may lead to a failure of R0 resection. Other later studies have rejected this opinion, suggesting that experienced surgeons can successfully perform a standard PPPD with complete removal of lesions and lymph nodes, leading to a successful R0 resection (4, 18). In conclusion, PPPD was a safe and effective surgical procedure for cancer in the pancreatic head in selected cases (4, 18).

Various suture techniques for pancreatojejunostomy have been introduced, but none of these techniques have been accepted as being optimal. The most popular techniques include end-to-side duct-to-mucosa anastomosis and end-to-end dunking or invagination anastomosis (20, 21); other methods were mostly modified from the above techniques (22–24). End-to-side duct-to-mucosa anastomosis is one of the most popular techniques, as it is thought as the most histologically compatible and has been reported to have excellent results in adults (5, 22, 25). Spagnoletti et al. performed PPPD and reported a successful end-to-side duct-to-mucosa anastomosis in a 5-month-old patient; the authors determined the method to be safe for pediatric patients (7). However, the authors also warned that the small MPD should be carefully managed during the suture in case of tearing the fragile tissue (7). Narrow MPD was a relative contraindication for duct-to-mucosa anastomosis (26). This technique is a complex procedure that requires sutures through the fragile tissue of the pancreas and has a high risk of tearing (27), especially in pediatric patients. Therefore, our team chose single-layer continuous sutures in the pancreatojejunostomy anastomosis with a stent to avoid tissue tearing and stenosis of the MPD. Our experience in end-to-end pancreatojejunostomy in pediatric patients may provide a reference for other surgeons who encounter similar cases.

Postoperative pancreatic fistula is the most challenging complication in PD. A pancreatic fistula is defined as any measurable volume of drain fluid on or after postoperative day 3 with an amylase level elevated to more than three times the upper limit of normal amylase (28). Our team sent the fluid to the anastomotic site for amylase examination every 3 days to exclude the pancreatic fistula. In our case, the patient recovered well without leak or fistula formation. Pancreatic leak and fistula formation are mostly related to the decrease in rate in the technique of pancreatic anastomosis and precise suturing of anastomoses by surgeons (21). In our experience, with the assistance of a modern robotic system, the three-dimensional field of vision made the anatomy of the pancreatic duct so clear that the anastomosis could be precisely sutured. However, no significant difference was found in the rate of pancreatic fistula between the robotic and open PD in recent studies (29–31). In addition to the suture technique, there are multiple factors responsible for pancreatic fistula formation after PD. Avoidance of pancreatic fistula has still a long way to go.

The robotic system used in the present study provided a wide three-dimensional field of vision, flexible tools, EndoWrist allowing seven degrees of freedom, and a steady traction without physiological tremor and allowed better control of hemostasis and precise dissection of tissues and hard sutures (32). After being practiced for more than a decade, the robotic approach is thought to be a major improvement over the traditional laparoscopic approach and could be applied to more complex procedures in a minimally invasive way (32). PD is widely accepted as one of the most complicated procedures in general surgery because of the wide dissection and three anastomoses involved. Robotic PD in adults has been reported to have better efficacy and safety (3, 25, 33, 34). Despite the reported advantages of robotic surgery, this approach is rarely used in pediatric surgery with only a few published reports (7–9). Our patient recovered well without complications, which proves robotic PD in pediatric patients to be safe and effective.

In conclusion, we presented here the first and youngest case of robotic PPPD for periampullary RMS in a pediatric patient. With the assistance of a modern robotic system, we performed an R0 resection and a child reconstruction with end-to-end pancreatojejunostomy, end-to-side hepaticojejunostomy, and duodenojejunostomy. Although DGE was found in our case, the patient recovered quickly within 3 weeks without fatal complications such as pancreatic fistula or leak. The case reported here demonstrates robotic PD in pediatric patients to be safe and effective without intra- or postoperative complications. However, further studies with longer follow-ups are required to evaluate clinical results.

## Data Availability

The raw data supporting the conclusions of this article will be made available by the authors, without undue reservation.
